# Expert recommendations on the management of hypertension in patients with ovarian and cervical cancer receiving bevacizumab in the UK

**DOI:** 10.1038/s41416-019-0481-y

**Published:** 2019-06-11

**Authors:** Chris Plummer, Agnieszka Michael, Ghazia Shaikh, Michael Stewart, Lynn Buckley, Tracie Miles, Agnes Ograbek, Terry McCormack

**Affiliations:** 1 Freeman Hospital, Newcastle upon Tyne, UK; 2 St Luke’s Cancer Centre, Royal Surrey County Hospital, Guildford, UK; 3 Northern Centre for Cancer Care, Newcastle upon Tyne, UK; 4 James Cook University Hospital, Middlesbrough, UK; 5 Hull and East Yorkshire Hospitals NHS Trust, Cottingham, UK; 6 Royal United Hospitals Bath NHS Foundation Trust, Bath, UK; 7 Roche Products Ltd., Welwyn Garden City, UK; 8 Hull York Medical School, York, UK

**Keywords:** Targeted therapies, Gynaecological cancer

## Abstract

Bevacizumab is an anti-vascular endothelial growth factor monoclonal antibody that may prolong survival in ovarian and cervical cancer when given in combination with chemotherapy. It works by blocking the signalling pathways that are required for tumour angiogenesis, potentially limiting the cancer’s ability to grow and spread. Hypertension is a known side effect of all angiogenesis inhibitors and could lead to interruption or premature discontinuation of effective anti-cancer treatment. Hypertension may also act as a barrier to the initiation of such treatment. In this review, we aim to present clear and practical recommendations on the management of blood pressure in ovarian and cervical cancer patients before, during and after bevacizumab treatment. This guidance covers considerations before initiating bevacizumab therapy and recommendations on the management of patients who develop hypertension, or who experience worsening of pre-existing hypertension, during bevacizumab treatment, and once the course of bevacizumab has been completed. These recommendations were developed collaboratively by a group of clinicians, comprising cardiologists, oncologists, a general practitioner and specialist oncology nurses, with expertise and practical experience in either oncology or hypertension. The aim of these recommendations is to support oncologists with hypertension assessment and management to facilitate starting or continuing bevacizumab.

## Background

Vascular endothelial growth factor (VEGF) plays a critical regulatory role in both normal and abnormal angiogenesis.^1^ It is essential for the vascular changes that occur during wound healing, ovulation and placental development, but also contributes to the development of new tumour vasculature in many human cancers.^1^ By binding to its receptors, VEGF activates complex intracellular signalling pathways that stimulate the proliferation, migration and survival of endothelial cells necessary for the generation of new blood vessels.^2^ By expressing VEGF continuously from the onset of development, tumours stimulate local angiogenesis and create their own blood supply from the existing vascular network.^3^ This ensures an ongoing source of essential nutrients and oxygen that is required for the continued growth of the tumour, and also facilitates intravasation and metastasis.^4,5^ Expression of VEGF has been shown to correlate with the biological aggressiveness of some tumours, including ovarian cancer, and is associated with poorer survival.^6,7^

### Bevacizumab and hypertension

Because of its role in tumour angiogenesis, VEGF has become an important target in cancer therapy and a number of anti-angiogenic inhibitors that block the VEGF pathway have been developed.^8–10^ These include those that target the VEGF ligand itself, such as bevacizumab, and several small molecule tyrosine kinase inhibitors (TKIs), such as sunitinib, sorafenib, cediranib, nintedanib and pazopanib, which block VEGF receptor functions.^10,11^ As with many other cancer treatments, VEGF inhibitors, particularly TKIs, have been associated with cardiovascular toxicity.^12–14^

Bevacizumab is a humanised monoclonal antibody that binds to all isoforms of VEGF.^9^ By binding to VEGF and preventing it from interacting with its receptors, bevacizumab inhibits angiogenesis and leads to the regression of newly formed microvessels and the ‘normalisation' of abnormal tumour vascularisation.^15,16^ Inhibition of VEGF is also directly related to the development of hypertension, a recognised class effect of anti-angiogenic therapies, including bevacizumab. As VEGF is needed to maintain the normal function of endothelial cells and vascular homoeostasis,^17,18^ blockade of the VEGF pathway can result in endothelial dysfunction and hypertension. The mechanism of this type of hypertension is not fully understood, but several theories have been proposed.^19–22^ One critical factor is thought to be a decrease in nitric oxide (NO) production, which occurs when VEGF is inhibited. Normally, binding of VEGF to its receptors on endothelial cells results in phosphorylation of the endothelial NO synthase enzyme and the production of NO, which diffuses to adjacent vascular smooth muscle cells.^23^ Here, NO triggers smooth muscle relaxation, resulting in vasodilation.^24^ VEGF is also involved in the production of other vasodilators, such as prostacyclin.^25^ It is thought that blocking the actions of VEGF by anti-angiogenesis inhibitors results in a deficiency in these vasodilators, a predominance of vasoconstrictive factors, such as endothelin-1, and ultimately an increase in blood pressure (BP).^24^ Plasma levels of endothelin-1 have been shown to increase after treatment with some anti-angiogenic therapies,^26,27^ leading to the suggestion that it may play a role in the development of on-treatment hypertension.^28,29^ Another proposed mechanism is the reduction in capillary bed density, or rarefaction, that may occur when VEGF is inhibited, leading to increased peripheral vascular resistance.^22^ Structural rarefaction has been demonstrated in the skin of bevacizumab-treated patients,^30^ but the extent of its role in the development of hypertension remains unknown.^28^ Decreases in NO in the kidney as a result of VEGF dysfunction can also lead to sodium retention and an increase in the extracellular fluid volume.^31^ This, together with systemic vasoconstriction caused by reduced vasodilators, increased vasoconstrictors and capillary rarefaction is likely to contribute to the development of hypertension during bevacizumab therapy.^31^

### Clinical trials of bevacizumab in gynaecological cancers

Bevacizumab is approved in Europe for the treatment of ovarian, fallopian tube and primary peritoneal cancer in combination with chemotherapy in the first-line and recurrent settings, and also for the treatment of persistent, recurrent or metastatic cervical cancer.^16^ A number of phase 3 clinical trials have assessed the efficacy of bevacizumab in combination with cytotoxic chemotherapy in the treatment of gynaecological cancers (Table 1).^32–38^ These trials demonstrated significant benefits in progression-free survival and overall survival in patients treated with bevacizumab compared with those not treated with bevacizumab.^33,36,38,39^ Patients with hypertension were not excluded from these trials unless the condition was uncontrolled, despite antihypertensive treatment. However, the proportion of patients with baseline hypertension was not generally reported, with the exception of two trials in which these patients made up 33–40% of the study population (Table 1).^32,35^

**Table 1:** Incidence of hypertension in pivotal phase 3 bevacizumab clinical trials in gynaecological cancers

	GOG-218^33^	ICON7^36^	ROSiA^35^	OCEANS^32,40^	GOG-213^34^	AURELIA^37^	GOG-240^38,39^
Indication	1L OC	1L OC	1L OC	Recurrent OC	Recurrent OC	Recurrent platinum-resistant OC	Recurrent, persistent or metastatic CC
Study design	Double-blind, Ph 3 RCT	Open-label, Ph 3 RCT	Single-arm, open-label, Ph 3b trial	Double-blind, Ph 3 RCT	Open-label, Ph 3 RCT	Open-label, Ph 3 RCT	Open-label, Ph 3 RCT
Assigned to bevacizumab, *n*	Initiation: 625 Throughout: 623	764	1 021	242	337	179	227
Bevacizumab given with	Paclitaxel/carboplatin	Paclitaxel/carboplatin	Paclitaxel/carboplatin	Gemcitabine/cisplatin	Paclitaxel/carboplatin	Single-agent chemotherapy	Cisplatin/paclitaxel or topotecan/paclitaxel
Median no. bevacizumab cycles (range)^a^	Initiation: 12 (0–22) Throughout: 14 (0–21)	17 (IQR 12–18)^b^	23 (1–61)	12 (1–43)	16 (1–111)	6 (1–24)	7 (0–36)
Baseline hypertension, *n* (%)	NR	NR	336 (32.9)	96 (39.7)	NR	NR	NR
Incidence of hypertension, *n* (%)							
All grades	NR	193 (25.9)	558 (54.7)	104 (42.1)	135 (40.9)	NR	NR
Grade ≥2	100 (16.5)/139 (22.9)	NR	NR	NR	NR	36 (20.1)	55 (25.0)
Grade ≥3	NR	46 (6.2)	252 (24.7)^c^	42 (17.0)	39 (11.8)	13 (7.3)	NR
Leading to discontinuation	NR	NR	30 (2.9)	10 (3.6)	NR	NR	NR

*1L* first-line, *CC* cervical cancer, *IQR* interquartile range, *NR* not reported, *OC* ovarian cancer, *RCT* randomised controlled trial

^a^Unless otherwise indicated. ^b^For women in the bevacizumab group who started chemotherapy >4 weeks after surgery. ^c^Includes six (0.6%) patients who experienced grade 4 hypertension

The development of on-treatment hypertension has been identified as a common adverse event in bevacizumab-treated patients, with an incidence of any grade hypertension of 26–55%^34–36,40^ and grade ≥3 hypertension of 6–25% (Table 1).^34–37,40^ The occurrence of bevacizumab-induced hypertension was more frequent during earlier cycles of treatment,^41^ but some cases have been reported following prolonged exposure to bevacizumab.^35^ In the ROSiA trial, which investigated an extended duration of frontline bevacizumab in patients with ovarian cancer, the median time to onset of hypertension was 2.1 months (range 0–28 months), with the majority (63%) of grade ≥3 hypertension occurring before 6 months.^35^ Grade 4 hypertension occurred in 6 (0.6%) patients in this study. In this trial and others in ovarian and cervical cancer, hypertension was typically manageable and discontinuations due to uncontrolled or symptomatic grade 3 hypertension, a pre-specified stopping point in most trials, were uncommon (Table 1).^35,40^ It should be noted that in classification of hypertension as a toxicity in clinical trials, the National Cancer Institute Common Terminology Criteria for Adverse Events (CTCAE) guidance is not aligned with current UK and European guidance on the diagnosis and treatment of hypertension (Table 2). CTCAE version 5.0 defines grade 3 hypertension as a BP ≥160/100 mmHg that requires antihypertensive treatment with one or more drugs.^42^ The majority of these trials used version 3.0 of the criteria, which did not use absolute BP as the primary determinant of the toxicity grading. For example, grade 3 toxicity was defined as hypertension ‘requiring more than one drug or more intensive therapy than previously' (Table 2). CTCAE grade 3 hypertension does not appear to fulfil the CTCAE definition for grade 3 toxicity of ‘severe or medically significant but not immediately life-threatening; hospitalisation or prolongation of hospitalisation indicated; disabling; limiting self-care [activities of daily life] ADL' in the same way as other grade 3 toxicities.^42^ CTCAE grade 3 hypertension would not necessarily be considered clinically significant in the primary care setting, and in most cases would be easily manageable. Grade 4 hypertension, however, is a medical emergency requiring immediate admission to a high-dependency unit for urgent monitoring and treatment. In all indications for which bevacizumab is licensed, cases of grade 4 hypertension have been rare, occurring in up to 1% of patients treated with bevacizumab plus chemotherapy versus up to 0.2% of patients treated with chemotherapy alone.^16^

**Table 2:** Comparison of hypertension grading/classification systems

Guidance	Blood pressure mmHg						Acute organ damage
	≥120/80	≥130/85	≥140/90	>150/100	≥160/100	≥180/110	
CTCAE v.3^76^	–			• Grade 1: asymptomatic transient increase • Grade 2: symptomatic recurrent/persistent increase requiring monotherapy • Grade 3: requiring >1 drug or more intensive therapy			Grade 4
CTCAE v.5^42^	Grade 1		Grade 2		Grade 3		Grade 4
NICE guidance^68^	Normal		Stage 1		Stage 2	Severe	Hypertensive emergency
ESH/ESC guidance^51^	Normal	High normal	Grade 1		Grade 2	Grade 3	Hypertensive emergency

*CTCAE* Common Terminology Criteria for Adverse Events, *ESC* European Society of Cardiology, *ESH* European Society of Hypertension, *NICE* National Institute for Health and Care Excellence

### Existing hypertension guidance

Several meta-analyses have demonstrated that bevacizumab increases the risk of hypertension in a range of solid tumours, including ovarian cancer,^43–46^ but there is a lack of specific guidance for oncologists on how to manage such patients. The National Institute for Health and Care Excellence (NICE) has published general guidance on the diagnosis and management of hypertension in adults, providing details of BP goals and treatment steps.^47^ This guidance was developed to improve public health and included a health economic assessment. Multiple clinical trials have shown that in the long-term, a persistently elevated BP increases the risk of cardiovascular events, such as stroke and myocardial infarction.^48^ Thus, reducing BP can reduce the occurrence and impact of these events in the general population.^49^

The NICE guidance categorises hypertension as stage 1 (BP ≥140/90 mmHg), stage 2 (BP ≥160/100 mmHg) or severe (systolic BP ≥180 mmHg or diastolic BP ≥110 mmHg).^47^ It recommends immediate treatment of patients with severe hypertension but initial repeat measurement, ideally with ambulatory or home BP monitoring (ABPM/HBPM), for those with stage 1 or 2 hypertension. For ABPM/HBPM, there are different diagnostic BP thresholds, ≥135/85 mmHg for stage 1 and ≥150/95 mmHg for stage 2. Drug treatment is only necessary in stage 1 if there are additional risk factors or evidence of vascular or target organ damage, whereas stage 2 always requires treatment.^47^ NICE recommends that antihypertensive medications are administered in a stepwise fashion.^50^ European hypertension guidelines are broadly similar to those from NICE, whereas the guidance from the Joint National Committee (JNC8) and the recently updated clinical practice guidelines from the American College of Cardiology/American Heart Association in the United States varies with respect to BP thresholds and management strategies.^51–53^ However, none of the guidance above is specific to bevacizumab-induced hypertension or an oncology patient population. While it is widely accepted that the long-term risks of hypertension are serious and warrant lifestyle and/or medical intervention, the short-term risk to an individual of a modest elevation in BP is low,^54^ unless there is an acute risk of accelerated phase or grade 4 hypertension. Patients who develop hypertension on bevacizumab are only exposed to an elevated BP for a relatively short duration with a mean treatment duration of ~13 months for the first-line treatment of ovarian cancer in routine practice.^55^ Furthermore, the life expectancy of cancer patients is lower than that of the general population; 5-year survival rates for patients with stage IV ovarian and cervical cancer are 4% and 5%, respectively.^56,57^ This presents a different scenario to that which the NICE guidelines, and others, aim to address, and so such guidelines are less meaningful in the oncology setting, where the risk of short-term hypertension has to be balanced against the survival advantages of active cancer treatments.

The manufacturers of bevacizumab recommend that pre-existing hypertension is adequately controlled before initiating bevacizumab therapy. In addition, they recommend permanently discontinuing the drug if hypertensive crisis or hypertensive encephalopathy develops, and in those with ‘medically significant hypertension' that cannot be adequately controlled with antihypertensive drugs.^16^ Further advice has been published to help oncologists who may not be familiar with managing hypertension in their clinical practice.^19,58–64^ Here, we focus on the management of bevacizumab-induced hypertension in cervical and ovarian cancer patients as this is the main area of prescribing for the VEGF inhibitor in the UK. This article aims to provide clear, practical guidance for the oncology team on managing patients with gynaecological cancer who experience hypertension during bevacizumab treatment, including considerations before initiation of therapy and once bevacizumab has been completed. Patients with significant pre-existing cardiovascular disease should have their overall cardiovascular risk assessed and their treatment optimised in consultation with their general practitioner and cardiologist.

## Methods

A consensus group of two cardiologists, two medical oncologists, a general practitioner (GP) and two specialist oncology nurses, all with experience in treating patients with bevacizumab and/or hypertension in clinical practice and trials, were invited by Roche to develop these recommendations. Initially, authors expressed their level of agreement with 27 statements that were developed based on a literature search of existing evidence and guidance (Table S1). PubMed was searched for existing advice on the management of hypertension during bevacizumab treatment using the terms (within title/abstract): Avastin, bevacizumab, anti-VEGF or VEGF inhibitor or angiogenesis inhibitor and hypertension or blood pressure and management, guideline, guidance, advice or consensus. There were no restrictions on publication date or language. This was followed by a face-to-face meeting to discuss areas of agreement/disagreement and develop pragmatic guidance for contemporary oncology practice.

## Recommendations

### Considerations before bevacizumab treatment

**Fig. 1:**
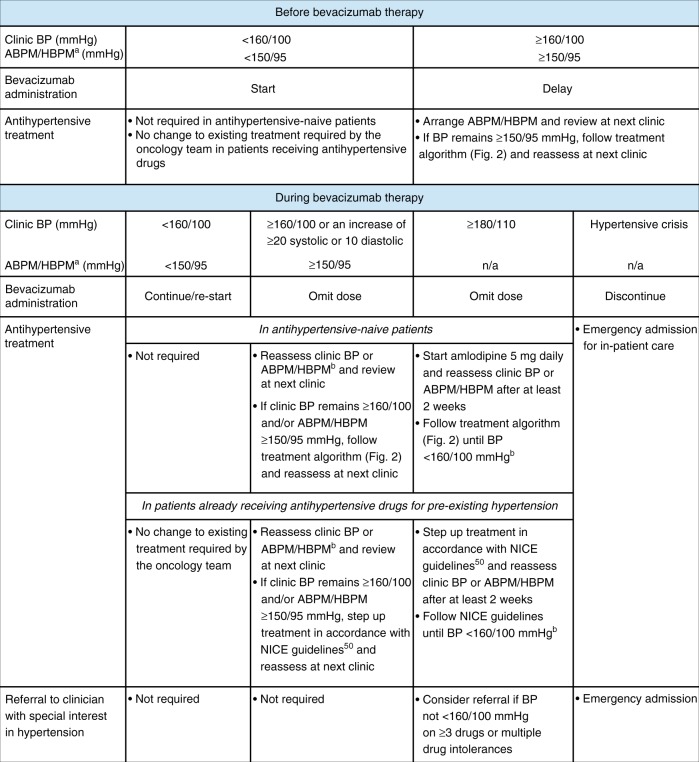
Management of hypertension before and during bevacizumab therapy. ^a^ABPM/HBPM recorded over at least 4 consecutive days. ^b^If there is a marked difference between clinic BP and ABPM/HBPM (i.e., >20/10 mmHg), the latter should be repeated with a treatment target of <150/95 mmHg. ABPM ambulatory blood pressure monitoring, BP blood pressure, HBPM home blood pressure monitoring, n/a not applicable, NICE National Institute for Health and Care Excellence

**Fig. 2:**
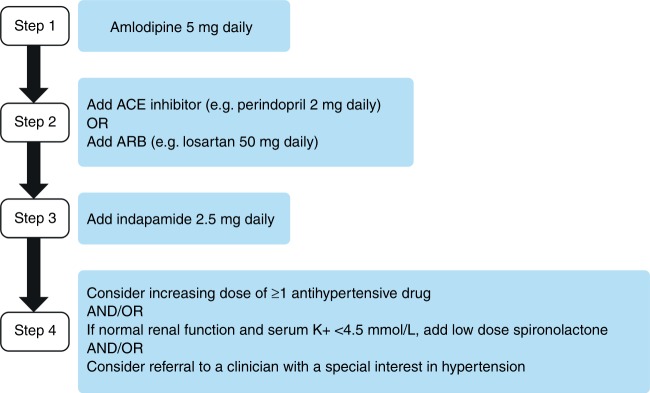
Treatment algorithm for antihypertensive-naive patients.^a^ Note: this algorithm is not appropriate for patients with significant pre-existing cardiovascular disease. ^a^If patient is already on antihypertensive treatment, manage in accordance with NICE hypertension guidelines.^47,50^ ACE angiotensin-converting enzyme, ARB angiotensin II receptor blocker, K+ potassium, NICE National Institute for Health and Care Excellence

It is a common practice for patients to have their BP assessed before beginning bevacizumab treatment. We recommend that bevacizumab can be started in all patients with a clinic BP <160/100 mmHg; initiation of new or additional antihypertensive treatment within the oncology unit is not required for these patients (Fig. 1). If the clinic BP is ≥160/100 mmHg, bevacizumab should not be started, and we recommend that ABPM or HBPM is arranged to confirm the level of sustained hypertension. When patients are asked to measure BP at home, this should be done in accordance with existing advice, such as that from NICE^65^ or the British and Irish Hypertension Society.^66^ If the average BP from ABPM or HBPM recorded over at least 4 consecutive days (ignoring day 1 measurements) is ≥150/95 mmHg, we recommend delaying the start of bevacizumab therapy. Antihypertensive treatment should be initiated with 5 mg amlodipine daily in antihypertensive-naive patients with patients being reassessed after at least 2 weeks (Figs. 1, 2). Amlodipine is considered a safe and efficient treatment for bevacizumab-associated hypertension.^67^ At the next visit, if BP from ABPM/HBPM is <150/95 mmHg, bevacizumab can be started. If BP from ABPM/HBPM remains ≥150/95 mmHg, patients should progress through the treatment algorithm (Fig. 2) until BP falls below this threshold, at which point bevacizumab administration can be initiated. Patients who are already receiving antihypertensive treatment should have their treatment stepped up in accordance with NICE guidance^50^ until clinic BP falls below 160/100 mmHg, at which point bevacizumab can be administered.^47^ When there is a discrepancy between clinic and ABPM/HBPM readings, i.e., one is in range and the other is not, clinical decisions should primarily be made on the ABPM/HBPM values as, in most circumstances, these represent a more reliable assessment of BP.^68^

We recommend that management of hypertension is initiated in the oncology unit whenever possible and then managed with the primary care team, who are best placed to monitor and treat the condition.^69^ Timely and effective communication between the oncology unit and the patient’s GP is essential for integrated care.^70,71^ The GP should be fully informed of the reason for initiating antihypertensive treatment and given specific instructions on treatment goals and the subsequent care that is required, i.e., GPs should follow the treatment algorithm until clinic BP reduces to <160/100 mmHg. This is typically done via a letter to the GP, but patients can be encouraged to play a key role in the shared care management plan by taking responsibility for such documents and handing them directly to their GP. Alternatively, this information could be captured within the patient’s chemotherapy record booklet or using a standard pro forma or BP measurement booklet, depending on local practice, and shared with the GP.

### Consideration during bevacizumab treatment

We recommend that BP is measured in all patients before each bevacizumab infusion. BP thresholds for the purposes of continuing bevacizumab remain the same as those set before starting bevacizumab treatment i.e., <160/100 mmHg. Patients who require antihypertensive treatment should be encouraged to measure their BP at home twice daily to monitor the effectiveness of this treatment.

If clinic BP before infusion of bevacizumab is <160/100 mmHg, bevacizumab can be given as normal. No antihypertensive treatment is required in antihypertensive-naive patients, and patients already on antihypertensive drugs should continue their current treatment (Fig. 1).

If clinic BP is ≥160/100 mmHg or there has been a marked increase of ≥20 mmHg systolic or ≥10 mmHg diastolic compared with previous assessments, we recommend that the bevacizumab dose is omitted and ABPM/HBPM is arranged. If the average BP from ABPM/HBPM is <150/95 mmHg, bevacizumab can be continued at the next scheduled clinic visit. If the BP remains ≥150/95 mmHg, antihypertensive-naive patients should be started on amlodipine 5 mg daily and those already on antihypertensive medication for pre-existing hypertension should have their treatment stepped up in accordance with NICE guidelines,^50^ and be reassessed after at least 2 weeks (Fig. 1). If clinic BP is <160/100 mmHg and/or ABPM/HBPM is <150/95 mmHg following initiation/step up of antihypertensive treatment, the patient can continue with the next dose of bevacizumab at the next scheduled clinic visit with concurrent antihypertensive treatment. If clinic BP remains ≥160/100 mmHg and/or ABPM/HBPM is ≥150/95 mmHg, the patient should step up antihypertensive treatment in accordance with the treatment algorithm shown in Fig. 2 (or in accordance with NICE guidance in those with pre-existing hypertension),^50^ and only restart bevacizumab once BP is <160/100 mmHg.

If a patient’s pre-bevacizumab infusion clinic BP is ≥180 systolic or ≥110 mmHg diastolic, we recommend that the dose of bevacizumab is omitted. Antihypertensive treatment should be initiated with amlodipine 5 mg daily in antihypertensive-naive patients (Fig. 2), or stepped up in accordance with NICE guidance in those already on antihypertensive therapy for pre-existing hypertension,^50^ and the patient should be reassessed with ABPM/HBPM after at least 2 weeks. If clinic BP falls to <160/100 mmHg with concurrent antihypertensive treatment, bevacizumab can be restarted. If there is a marked difference between clinic BP and ABPM/HBPM (>20/10 mmHg), the latter should be repeated with a treatment target of <150/95 mmHg. If BP fails to drop below these thresholds despite treatment with ≥3 antihypertensive drugs, or if there are multiple drug intolerances, consider referral to a clinician with a special interest in hypertension for advice.

If an antihypertensive-naive patient presents with an excessively high BP i.e., ≥220 mmHg systolic, they should be checked for evidence of functional deterioration of vital organs, such as that seen in hypertensive crises. If there is no decompensation, we recommend that the patient is treated with amlodipine 5 mg daily and reassessed in 2 weeks. Bevacizumab should be permanently withheld in all patients who develop malignant-phase hypertension, hypertensive crisis or hypertensive encephalopathy, and emergency referral should be arranged for in-patient treatment.

A patient’s GP should be informed via letter of the development of hypertension during bevacizumab therapy and of any antihypertensive treatment required. We recommend that the GP is given clear, specific instructions for the patient’s ongoing care and BP targets i.e., the patient should follow the treatment algorithm until BP reduces to <160/100 mmHg. The ongoing care of the patient should be shared between the oncology unit and primary care.^71^ Communication between a patient’s oncologist and GP is essential for optimal integrated management, and the patient should be encouraged to play a key role within this shared management plan by carrying documents relating to their care to present at GP appointments.^70,72^

### Considerations after the course of bevacizumab is complete

Hypertension that develops during bevacizumab therapy typically resolves after the treatment course of bevacizumab is complete. One study in gynaecological cancers found that hypertension resolved in 28 out of 34 patients after a median of 87 (range 3–236) days after the last dose of bevaciziumab.^73^ We recommend that all patients who develop on-treatment hypertension requiring new antihypertensive treatment are encouraged to arrange follow-up in primary care within 4 weeks of stopping bevacizumab to have their BP re-measured. The need for ongoing antihypertensive medication should be reassessed and a plan for reducing/stopping antihypertensive treatment put in place, if necessary, to ensure patients do not develop hypotension. Once the patient’s BP has returned to normal, we recommend that BP be monitored annually.

## Conclusions

The aim of these recommendations is to provide practical advice to oncologists who may be unfamiliar with dealing with hypertension. Pre-existing hypertension with a BP <160/100 mmHg should not be a barrier to starting bevacizumab. Similarly, as the short-term absolute risk of a moderately elevated BP is low, a BP <160/100 mmHg does not require suspension or discontinuation of bevacizumab or the initiation/up-titration of antihypertensive treatment. This threshold is used for the purpose of deciding whether a patient can initiate or continue bevacizumab treatment and is separate to the NICE BP target of <140/90 mmHg, which remains the long-term goal in patients with hypertension. The management of these patients with bevacizumab-induced hypertension should be shared between the oncology unit and primary care. Good communication, including specific instructions and treatment goals, is vital for the success of such a shared management plan.

These recommendations have been developed for bevacizumab treatment, but hypertension is common with many anti-angiogenic drugs, including small molecule tyrosine kinase inhibitors.^74^ Although the incidence and relative risk of hypertension varies between drugs,^75^ the mechanisms are likely to be the same. The principles behind these recommendations, that the short-term risk of an elevated BP is low and should be balanced against the risk of interrupting evidence-based anti-cancer therapy, are also relevant to other anti-angiogenic drugs. We postulate that the recommendations set out above are generic and applicable to other drugs in this class. We also suggest that, although we have focused on bevacizumab use in gynaecological cancers, the recommendations could be extrapolated to other cancer indications.

## Supplementary information

Supplemental material

## Data Availability

Data sharing is not applicable to this article as no datasets were generated or analysed during the current study.
