# Supplementary material for: Expert recommendations on the management of hypertension in patients with ovarian and cervical cancer receiving bevacizumab in the UK
Source: Br J Cancer. 2019 Jun 11;121(2):109–16. doi: 10.1038/s41416-019-0481-y (PMC6738076; doi:10.1038/s41416-019-0481-y)
Supplement: Supplementary file 1 — Supplemental material [file 41416_2019_481_MOESM1_ESM.docx]

# Supplementary information for:

# Expert Recommendations on the Management of Hypertension in Patients with Ovarian and Cervical Cancer Receiving Bevacizumab in the UK

**Running title:** Bevacizumab and hypertension expert recommendations

Chris Plummer,^1*^ Agnieszka Michael,^2^ Ghazia Shaikh,^3^ Michael Stewart,^4^ Lynn Buckley,^5^ Tracie Miles,^6^ Agnes Ograbek,^7^ Terry McCormack^8^

*^1^Freeman Hospital, Newcastle upon Tyne, UK; ^2^St Luke’s Cancer Centre, Guildford, UK; ^3^Northern Centre for Cancer Care, Newcastle upon Tyne, UK; ^4^James Cook University Hospital, Middlesbrough, UK; ^5^Hull and East Yorkshire Hospitals NHS Trust, Cottingham, UK; ^6^Royal United Hospitals Bath NHS Foundation Trust, Bath, UK; ^7^Roche Products Ltd., Welwyn Garden City, UK; ^8^Hull York Medical School, York, UK*

**^*^Corresponding author:** Dr Chris Plummer, Consultant Cardiologist, Freeman Hospital, Freeman Road, High Heaton, Newcastle upon Tyne, NE7 7DN. Tel: +44 (0)191 2336161. Email: [Chris.Plummer@nhs.net](mailto:Chris.Plummer@nhs.net)

Table S1. Survey questions/statements.

| **General** | |
| --- | --- |
| 1 | Are you familiar with the [current NICE guidelines](https://www.nice.org.uk/guidance/cg127/chapter/1-Guidance) on the management of hypertension? |
| **Before starting bevacizumab treatment** | |
| 2 | Target blood pressure before starting bevacizumab therapy should mirror the recommendations in the NICE guidelines on management of hypertension in the general population. |
| 3 | Patients with pre-existing hypertension should have their condition controlled with antihypertensive treatment before starting bevacizumab. |
| 4 | A patient’s GP should be informed of the reason for initiating antihypertensive treatment and the target blood pressure. |
| **During bevacizumab treatment** | |
| 5 | Blood pressure should be measured in all patients before and after each bevacizumab infusion at every cycle during treatment. |
| 6 | More frequent blood pressure assessments are required if bevacizumab-induced hypertension develops. |
| 7 | Blood pressure goals remain the same as those set before starting bevacizumab treatment i.e. in line with NICE guidelines (<140/90 mmHg in patients under 80 years of age, <150/90 mmHg in those aged over 80 years of age, etc). |
| 8 | Higher blood pressure ranges are acceptable during bevacizumab therapy. |
| 9 | The risks of an elevated blood pressure in the short-term are low for bevacizumab-treated patients. |
| 10 | Prompt intervention is vital when bevacizumab-induced hypertension develops to avoid dose reductions or discontinuations. |
| 11 | Bevacizumab should be continued in patients who develop stage 1 hypertension (≥140/90 mmHg) during bevacizumab treatment. |
| 12 | Bevacizumab should be continued in patients who develop stage 2 hypertension (≥160/100 mmHg) during bevacizumab treatment. |
| 13 | Antihypertensive treatment should be offered to all patients who develop stage 1 hypertension (≥140/90 mmHg) on bevacizumab treatment. |
| 14 | Antihypertensive treatment should be offered to all patients who develop stage 2 hypertension (≥160/100 mmHg) on bevacizumab treatment. |
| 15 | When treatment for bevacizumab-induced hypertension is indicated, it should be given in accordance with [NICE guidelines](https://pathways.nice.org.uk/pathways/hypertension#path=view%3A/pathways/hypertension/treatment-steps-for-hypertension.xml&content=view-index). Please note any exceptions in the text box. |
| 16 | In patients with severe bevacizumab-induced hypertension (≥180 systolic or ≥110 mmHg diastolic), bevacizumab should be suspended, and the patient referred for specialist management. |
| 17 | In patients with severe bevacizumab-induced hypertension (≥180 systolic or ≥110 mmHg diastolic), bevacizumab can be restarted once blood pressure reaches below 160/100 mmHg with concurrent antihypertensive treatment. |
| 18 | Bevacizumab should be permanently withheld in patients who develop malignant phase hypertension, hypertensive crisis or hypertensive encephalopathy. |
| 19 | Patients with pre-existing hypertension who develop bevacizumab-induced hypertension should be treated in the same way as those with no previous history of hypertension. |
| 20 | A patient’s GP should be informed via letter of the development of bevacizumab-induced hypertension, the treatment received by the patient and the short- and long-term blood pressure goals. |
| 21 | The GP should be responsible for the ongoing management of a patient’s hypertension throughout bevacizumab treatment, with support from oncology team and specialist advice if required. |
| 22 | Ongoing communication between a patient’s oncologist and GP is essential for the optimal management of bevacizumab-induced hypertension. |
| **After bevacizumab treatment** | |
| 23 | Bevacizumab-induced hypertension resolves after completion of bevacizumab therapy. |
| 24 | The need for ongoing antihypertensive medication should be reassessed once bevacizumab treatment has stopped, and a plan for reducing/stopping antihypertensives put in place, if necessary. |
| 25 | The GP is responsible for titrating down the antihypertensive medication with guidance from the oncology team. |
| 26 | The blood pressure of patients who have developed bevacizumab-induced hypertension should be monitored more frequently after completion of bevacizumab therapy than in those who do not. |
| **Other** | |
| 27 | Are there other areas of concern relating to the management of bevacizumab-induced hypertension that you feel need to be addressed? |

Survey respondents were asked to consider their specific experiences with managing patients with ovarian or cervical cancer when responding to the questions/statements. Respondents were able to indicate how much they agreed/disagreed with each statement using a 7-point scale, with additional free-text space to add comments or elaborate on the scoring.

GP, general practitioner; NICE, National Institute for Health and Care Excellence.
